# Transaxillary Versus Transfemoral Access for Transcatheter Aortic Valve Implantation: A Propensity Score-Matched Comparison of Clinical Outcomes Using VARC-3 Endpoints

**DOI:** 10.3390/diagnostics16172855

**Published:** 2026-09-05

**Authors:** Kemal Eşref Erdoğan, Emrah Uğuz, Mehmet Akif Erdöl, Muhammet Fethi Sağlam, Murat Yücel, Altay Alili, Hüseyin Bayram, Mehmet Murat Yiğitbaşı, Burak Kardeşler, Kamuran Kalkan

**Affiliations:** 1 Department of Cardiovascular Surgery, Ankara Yildirim Beyazit University, School of Medicine and Ankara Bilkent City Hospital, Ankara 06800, Türkiye; emrahuguz@gmail.com (E.U.); dr.m.fethisaglam@gmail.com (M.F.S.); 2 Department of Cardiology University of Health Sciences, Faculty of Medicine, Ankara Bilkent City Hospital, Ankara 06800, Türkiye; akiferdol@gmail.com (M.A.E.); drhuseyinbayram@gmail.com (H.B.); 3 Department of Cardiovascular Surgery, Ankara Bilkent City Hospital, Ankara 06800, Türkiye; dr_yucelmurat@hotmail.com (M.Y.); alilialtay97@gmail.com (A.A.); 4 Department of Cardiology, Ankara Yildirim Beyazit University, School of Medicine and Ankara Bilkent City Hospital, Ankara 06800, Türkiye; mmuratyigitbasi@gmail.com (M.M.Y.); kamurankalkandr@gmail.com (K.K.); 5 Department of Cardiology, Ankara Bilkent City Hospital, Ankara 06800, Türkiye; burak_kardesler@hotmail.com

**Keywords:** transcatheter aortic valve implantation, transaxillary access, transfemoral access, propensity score matching, VARC-3, bleeding complications, alternative access

## Abstract

**Background/Objectives**: Transaxillary access (TAx) is an established alternative to transfemoral access (TF) for transcatheter aortic valve implantation (TAVI) in patients with unfavorable iliofemoral anatomy. While TAx-TAVI has been increasingly adopted at experienced centers, propensity score-matched comparative data with VARC-3 endpoint definitions are scarce, and most existing series originate from Western European cohorts. We aimed to address this gap by reporting outcomes from a propensity-matched TAx versus TF cohort using VARC-3 endpoints, contributing contemporary data from a high-volume Turkish center with established alternative access expertise. **Methods**: Among 2389 consecutive TAVI procedures screened between January 2016 and December 2024, 291 patients with complete data were included. After stratification by access route (TAx *n* = 51; TF n = 240) and exclusion of one TAx patient with missing covariates, 1:2 greedy nearest-neighbor propensity score matching was performed using nine covariates. The final cohort comprised 150 patients (50 TAx, 100 TF). All outcomes were defined per VARC-3 criteria. **Results**: All nine covariates achieved standardized mean differences <0.1 after matching. Technical success was comparable (TAx 94.0% vs. TF 96.0%; *p* = 0.686). The VARC-3 early safety event rate did not differ significantly (16.0% vs. 15.0%; *p* = 1.000); no equivalence can be inferred given the small event counts and wide confidence intervals. Any bleeding (VARC-3) was significantly lower in the TAx group (8.0% vs. 22.0%; OR 0.31, 95% CI 0.10–0.95; *p* = 0.039), confirmed by conditional logistic regression for the matched structure (OR 0.32 [0.10–0.97]; *p* = 0.043); this should be considered an exploratory finding. Thirty-day all-cause mortality (encompassing in-hospital deaths) (6.0% vs. 10.0%; *p* = 0.545), 1-year mortality (12.0% vs. 16.0%; *p* = 0.628), and Kaplan–Meier 1-year survival (88.0% vs. 84.0%; log-rank *p* = 0.510) did not differ significantly. ICU stay (median 2 [1–2] vs. 2 [1–3] days; *p* = 0.020) and hospital stay (median 5 [4–6] vs. 5 [4–6] days; *p* = 0.005) yielded statistically significant *p*-values; however, medians were identical in both comparisons, and significance was driven by outlier-prolonged admissions in the TF group rather than a meaningful difference in typical recovery duration. **Conclusions**: TAx-TAVI demonstrated comparable mortality, safety, and hemodynamic outcomes to TF-TAVI, with a significantly lower rate of bleeding complications. Axillary access represents a safe and effective alternative route in patients unsuitable for transfemoral TAVI.

## 1. Introduction

Transcatheter aortic valve implantation has become the standard of care for symptomatic severe aortic stenosis across the full spectrum of surgical risk, as established by current European and American guidelines [1,2]. The transfemoral approach remains the preferred default access route owing to its established safety profile, feasibility under local anesthesia, and association with lower vascular complication rates and shorter procedural times in anatomically favorable candidates, as demonstrated in landmark randomized trials [3,4]. Nevertheless, a clinically significant proportion of TAVI candidates—estimated at 15–20%—present with unfavorable iliofemoral anatomy precluding the transfemoral route [5]. Common transfemoral contraindications include severe iliofemoral calcification, inadequate vessel caliber (diameter < 5.5 mm), and significant tortuosity. Our institution has previously characterized the predictors of vascular complications in the transfemoral TAVI setting, identifying vessel dimensions, calcification burden, and sheath-to-artery ratio as key determinants—findings that subsequently guided the development of our institutional access route selection criteria [6].

Among available non-femoral alternatives, the axillary artery approach has emerged as one of the most widely adopted at experienced centers [7,8]. The distal subclavian–proximal axillary artery segment provides a direct, anatomically predictable trajectory to the aortic valve, accommodates both self-expanding and balloon-expandable valve systems, and is amenable to surgical cutdown under local anesthesia with conscious sedation. Our center has progressively built expertise across the spectrum of alternative access TAVI: we reported the first transaxillary implantation experience with a Meril balloon-expandable system [9], and subsequently demonstrated the feasibility of transcarotid TAVI with the Myval device under local anesthesia [10]. The present study represents a systematic, propensity-matched evaluation of our axillary TAVI experience against the transfemoral standard.

The Valve Academic Research Consortium-3 (VARC-3) consensus document, published in 2021, established updated and harmonized endpoint definitions for aortic valve interventional research [11]. The VARC-3 early safety event composite—encompassing 30-day all-cause mortality, stroke, bleeding ≥ Type 2, acute kidney injury >Stage 2, major vascular complication, major cardiac structural complication, valve embolization, and vascular reintervention—provides a clinically meaningful, multidimensional measure of early procedural safety. Most published TAx versus TF comparisons predate VARC-3 or apply heterogeneous endpoint definitions, limiting cross-study comparability.

Existing meta-analyses and registry analyses comparing axillary and transfemoral TAVI have generally demonstrated comparable mortality outcomes, with some evidence of lower vascular complication rates with axillary access [7,12]. However, most studies are underpowered for rare endpoints, lack propensity score adjustment, or derive from Western European populations that may not fully represent the comorbidity profile encountered at centers in Turkey and the broader region. In the present study, we address these gaps by reporting outcomes from a propensity score-matched cohort with VARC-3 endpoint computation performed de novo from individual component variables.

## 2. Materials and Methods

### 2.1. Study Design and Population

**Figure 1:**
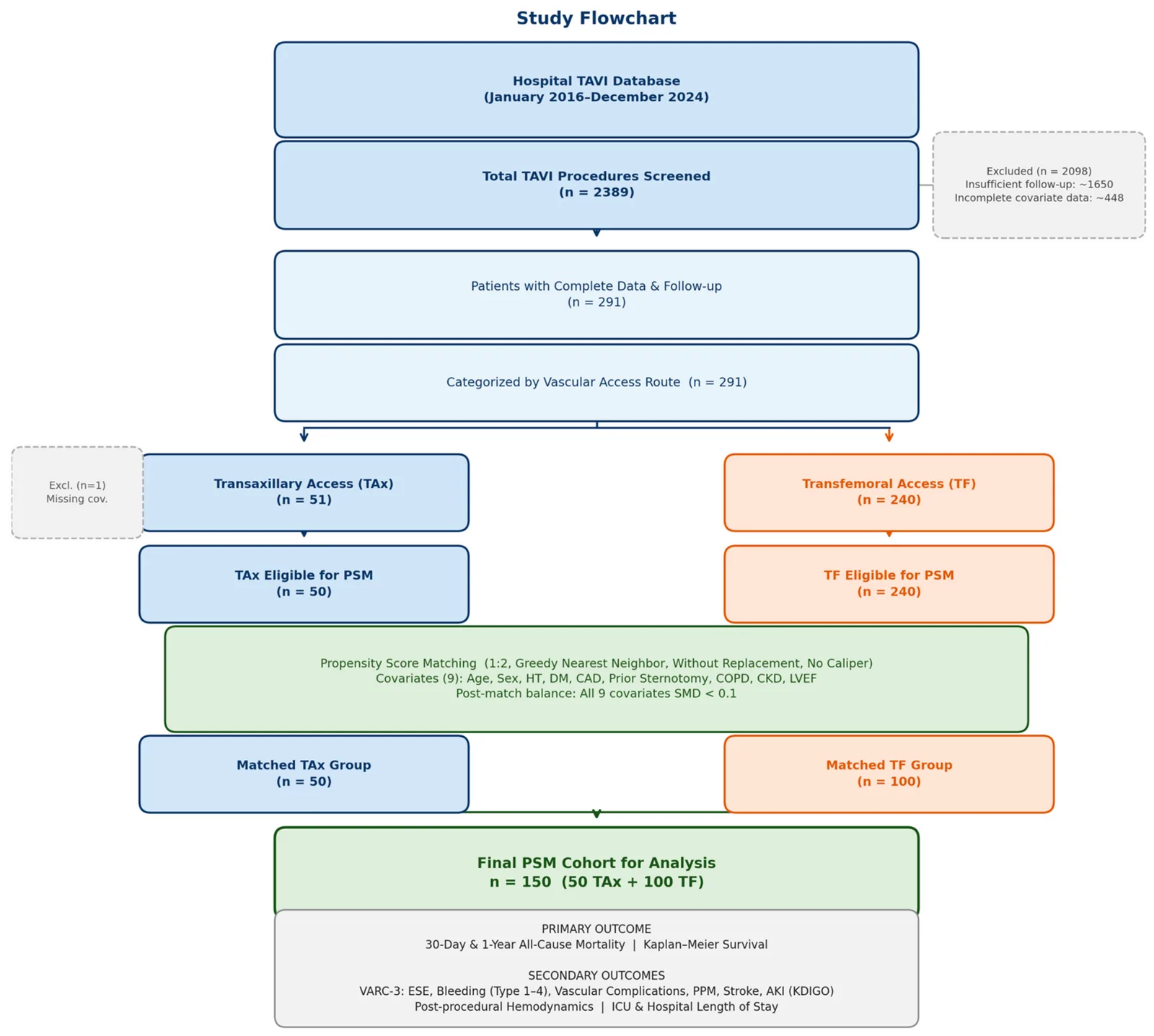
Study flowchart. Among 2389 TAVI procedures screened (January 2016–December 2024), 291 patients with complete data and follow-up were included. After stratification by access route and exclusion of one TAx patient with missing propensity score covariates, 1:2 greedy nearest-neighbor propensity score matching yielded a final cohort of 150 patients (50 TAx, 100 TF). TAx, transaxillary access; TF, transfemoral access; PSM, propensity score matching; VARC-3, Valve Academic Research Consortium-3.

This was a retrospective, single-center, propensity score-matched cohort study. Consecutive patients undergoing TAVI at our institution between January 2016 and December 2024 were identified from a prospectively maintained institutional database. Among 2389 TAVI procedures screened, 2098 patients were excluded due to incomplete clinical data or insufficient follow-up, yielding 291 patients with complete datasets. Patients were stratified by vascular access route: transaxillary access (TAx; n = 51) and transfemoral access (TF; n = 240). One TAx patient was excluded due to missing propensity score covariates, resulting in 50 TAx patients eligible for matching (Figure 1). The study was conducted in accordance with the Declaration of Helsinki and approved by the institutional Clinical Research Ethics Committee (Approval No. E2-23-3200). Informed consent was waived given the retrospective design.

### 2.2. Access Route Selection and Procedural Technique

Access route selection was determined by the multidisciplinary Heart Team following preprocedural multidetector computed tomographic angiography (MDCT). Transfemoral access was the preferred default approach. Axillary access was selected in patients with unfavorable iliofemoral anatomy, defined as common femoral or iliac artery diameter < 5.5 mm, heavy calcifications, or significant tortuosity precluding safe transfemoral sheath delivery. The diameter, tortuosity, and calcium distribution of the axillary and subclavian arteries were analyzed on MDCT with particular attention to the subclavian origin from the aortic arch. A patent left internal mammary artery (LIMA) graft was not regarded as an absolute contraindication to the axillary approach, provided that the vessel diameter exceeded 7 mm and was free of significant atherosclerotic disease; MDCT-based measurement of the distance from the LIMA ostium to the planned access site guided this decision in all such cases. The presence of a permanent pacemaker or implantable cardioverter–defibrillator (ICD) likewise did not constitute an absolute contraindication. Patients with unsuitable axillary anatomy were referred for carotid, brachiocephalic, or direct aortic approaches and were excluded from this analysis.

In our practice, the left axillary artery was preferred in all eligible patients. The axillary artery was accessed through a surgical cutdown, dissecting the vessel free over several centimeters with vessel loops placed proximally and distally to achieve vascular control. Hemostasis was secured using a 5/0 polypropylene pledgeted purse-string suture technique, through which an access needle and a J-tipped guidewire were sequentially inserted. The purse-string technique was consistently applied at vessel closure immediately following sheath removal, ensuring reliable hemostasis and avoiding significant post-procedural access-site bleeding. Pulse oximetry of the ipsilateral hand was confirmed before wound closure to verify distal arm perfusion. Both self-expanding (CoreValve, Medtronic, Minneapolis, USA; Acurate, Boston Scientific, Marlborough, USA) and balloon-expandable (SAPIEN, Edwards Lifesciences, Irvine, USA; Myval Meril Life Sciences, Vapi, India) valve systems were deployed across the study period according to Heart Team consensus and anatomical suitability [9,10].

### 2.3. Propensity Score Matching

**Figure 2:**
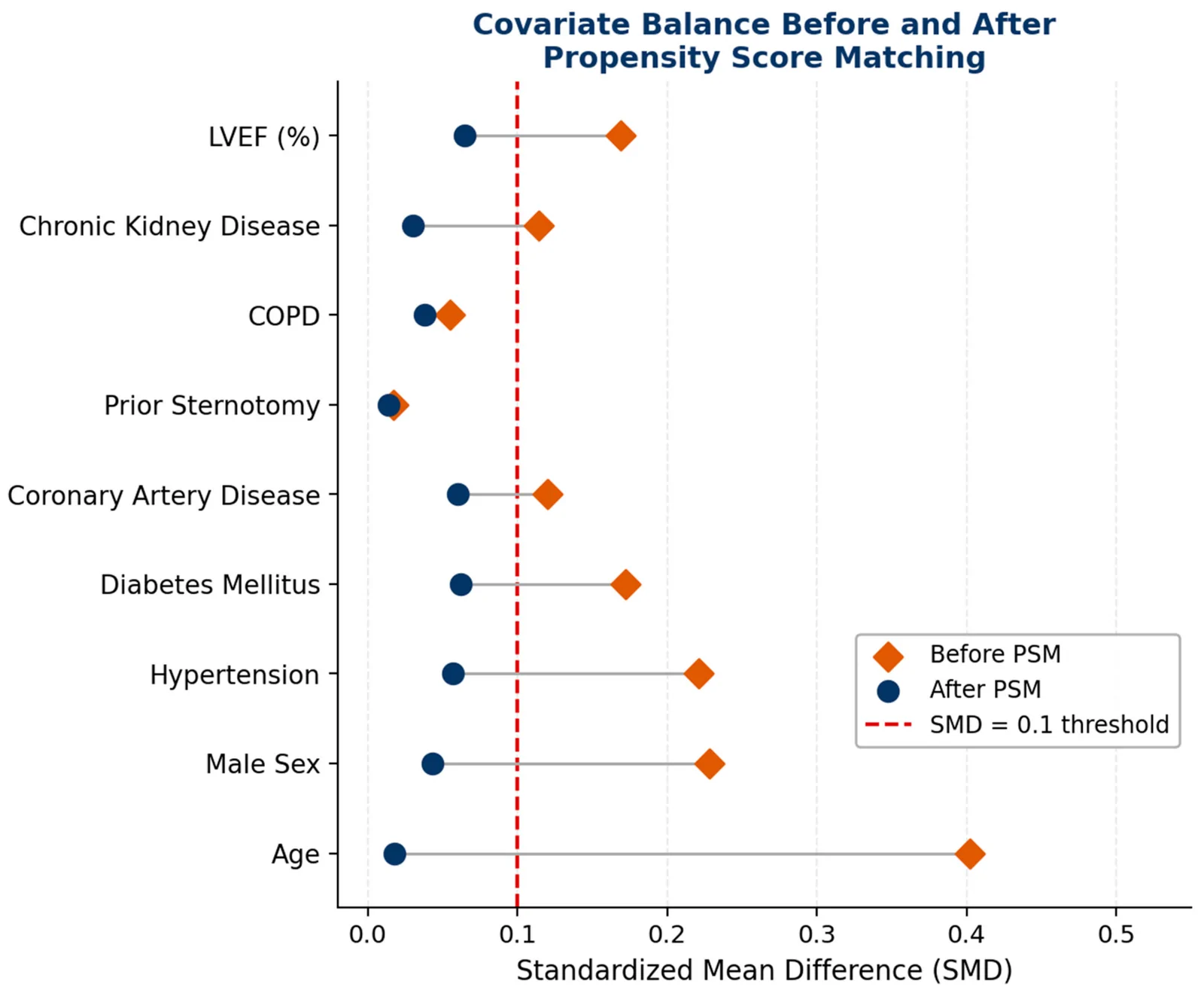
Love plot showing standardized mean differences (SMD) for nine propensity score covariates before (orange diamonds) and after (blue circles) matching. The dashed vertical line indicates the SMD = 0.1 adequacy threshold. All nine covariates achieved SMD < 0.1 after matching. DM, diabetes mellitus; HT, hypertension; CAD, coronary artery disease; COPD, chronic obstructive pulmonary disease; CKD, chronic kidney disease; LVEF, left ventricular ejection fraction.

Propensity scores were estimated using multivariable logistic regression with nine standardized preoperative covariates: age, sex, hypertension, diabetes mellitus, coronary artery disease, prior sternotomy, chronic obstructive pulmonary disease, chronic kidney disease, and left ventricular ejection fraction. Variables mechanistically linked to access route selection (peripheral artery disease, porcelain aorta, aortic valve area) were deliberately excluded to avoid indication-related over-adjustment [13]. Specifically, peripheral artery disease was the primary indication for TAx access in 74% of the TAx cohort; including this variable in the propensity model would constitute indication confounding by adjusting for the very factor determining treatment assignment rather than a neutral covariate. STS Score was likewise excluded, as its marked elevation in the TAx group (8.8 ± 3.2% vs. 3.8 ± 2.4%; *p* < 0.001) reflects the aggregated comorbidity burden (peripheral vasculopathy, prior sternotomy, multimorbidity) characteristic of patients referred for alternative access—making STS an access-selection variable rather than a neutral confounder. A 1:2 greedy nearest-neighbor matching algorithm without replacement and without caliper restriction was applied. Covariate balance was assessed using standardized mean differences (SMD); SMD < 0.1 was defined a priori as adequate balance [14]. All nine covariates achieved SMD < 0.1 after matching (Figure 2). Propensity score distributions and common support were assessed graphically (Supplementary Table S1). As a pre-specified sensitivity analysis, conditional logistic regression (CLR) was performed for all binary outcomes using Match ID as the cluster identifier, to account for the matched 1:2 structure.

### 2.4. Outcome Definitions

All outcomes were defined according to VARC-3 [11]. Primary outcomes were 30-day and 1-year all-cause mortality. Secondary outcomes included technical success, device success, ESE (composite as defined above), new permanent pacemaker implantation, moderate-to-severe paravalvular aortic regurgitation, stroke, neurological dysfunction without CNS injury, acute kidney injury > Stage 2, procedural myocardial infarction, any bleeding (VARC-3), vascular complications, vascular reintervention, procedure-related rehospitalization, and post-procedural hemodynamics. The VARC-3 ESE composite was computed de novo from individual component variables to ensure VARC-3 definitional compliance and eliminate potential pre-recorded composite miscategorization.

Acute kidney injury was staged according to the Kidney Disease: Improving Global Outcomes (KDIGO) criteria as incorporated in the VARC-3 consensus document [11]: Stage 1, serum creatinine increase ≥1.5× baseline or ≥0.3 mg/dL within 48 h; Stage 2, ≥2.0× baseline; Stage 3, ≥3.0× baseline, initiation of renal replacement therapy, or estimated GFR < 35 mL/min/1.73 m^2^. The pre-specified threshold was AKI > Stage 2, consistent with the VARC-3 ESE definition. VARC-3 endpoint definitions were retrospectively applied to all events across the study period using prospectively collected source data. Independent adjudication by a formal clinical events committee was not performed given the retrospective single-center design; this represents a methodological limitation, as VARC-3 was published in 2021 and events from 2016–2020 were reclassified retrospectively. The ESE composite was computed de novo from individual component variables to minimize misclassification risk.

### 2.5. Statistical Analysis

Continuous variables are presented as mean ± standard deviation or median [interquartile range] and compared using the Mann–Whitney U test. Categorical variables are presented as frequencies and percentages and compared using Fisher’s exact test with unmatched four-fold-table odds ratios. For cells with zero events, the Haldane–Anscombe correction (addition of 0.5 to all cells) was applied. As a pre-specified sensitivity analysis to account for the matched 1:2 structure, conditional logistic regression (CLR) was performed for all binary outcomes using the Match ID as the cluster identifier. Kaplan–Meier analysis with log-rank testing was used for time-to-event outcomes. A two-tailed *p* < 0.05 was considered statistically significant. Given the exploratory nature of multiple secondary comparisons, no adjustment for multiplicity was applied; all secondary findings should be interpreted as hypothesis-generating and not as confirmatory evidence. All analyses were conducted in Python 3.12 (statsmodels 0.14, scikit-learn 1.3, scipy 1.11, lifelines 0.27).

## 3. Results

### 3.1. Baseline Characteristics

After 1:2 propensity score matching, the final cohort comprised 50 patients in the TAx group and 100 patients in the TF group. Baseline characteristics are presented in Table 1. All nine matched covariates achieved SMD < 0.1 after matching, with the largest post-match SMD being 0.074 for hypertension, confirming excellent group comparability. Mean age was similar between groups (79.0 ± 5.8 years in TAx vs. 79.1 ± 5.4 years in TF; *p* = 0.865), as was the proportion of male patients (54.0% vs. 56.0%; *p* = 0.863). Prior sternotomy was present in 32.0% of patients in both groups (*p* = 1.000).

As anticipated from the indication-based access route selection process, the TAx group exhibited substantially higher rates of peripheral artery disease (74.0% vs. 24.0%; *p* < 0.001) and higher STS risk scores (8.8 ± 3.2% vs. 3.8 ± 2.4%; *p* < 0.001). These differences reflect the underlying anatomical and clinical profile that precluded transfemoral access in the TAx patients and are reported for clinical context rather than as residual confounders.

### 3.2. Procedural Characteristics

Procedural characteristics are presented in Table 2. Balloon-expandable valves were used more frequently in the TAx group (64.0% vs. 39.0%; *p* = 0.005), reflecting institutional practice and the Heart Team’s preference for balloon-expandable systems via axillary access due to their precise deployment characteristics in the axillary trajectory [9,10]. Local anesthesia was used in 78.0% of TAx procedures versus 95.0% of TF procedures (*p* = 0.003), a difference that reflects the institutional adoption of local anesthesia for TAx-TAVI over the 2016–2024 study period, as discussed further in Section 4. Mean sheath size was larger in the TAx group (15.9 ± 1.8 F vs. 14.7 ± 1.0 F; *p* < 0.001), consistent with the technical requirements of the axillary surgical approach.

**Figure 3:**
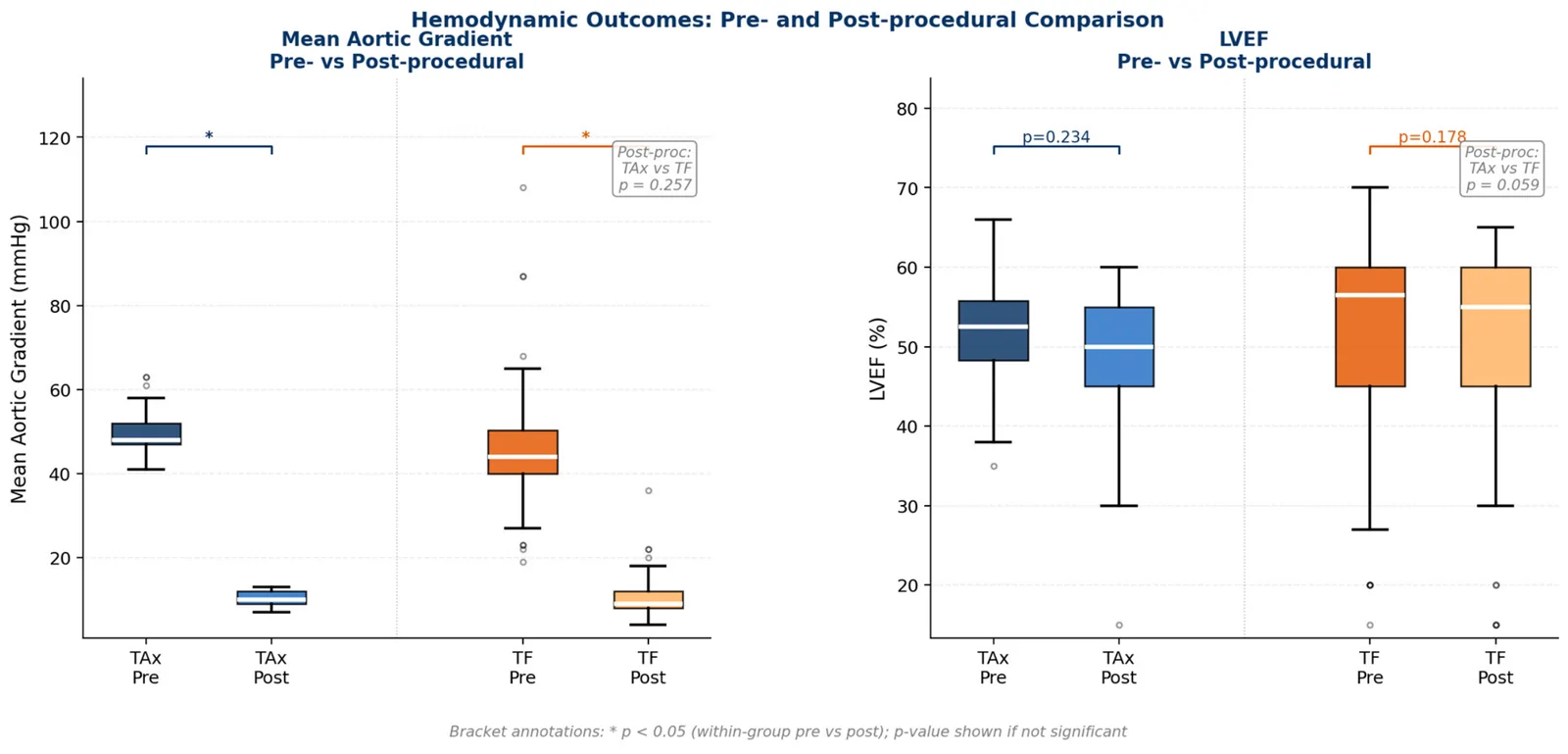
Box plots showing pre- and post-procedural mean aortic gradient (mmHg; **left panel**) and left ventricular ejection fraction (LVEF, %; **right panel**) in the TAx and TF groups. Dark blue and light blue boxes represent TAx pre- and post-procedural values, respectively; orange and light orange boxes represent TF values. Bracket annotations indicate within-group pre-to-post significance (* *p* < 0.05; *p*-value displayed if not significant). Post-procedural between-group comparison (TAx vs. TF) is shown in the upper right corner of each panel. LVEF, left ventricular ejection fraction.

Post-procedural hemodynamic outcomes were comparable between groups. Mean aortic gradient decreased significantly in both groups following the procedure (TAx: 49.3 ± 14.0 to 10.3 ± 2.0 mmHg, *p* < 0.001; TF: 46.0 ± 15.2 to 10.4 ± 4.3 mmHg, *p* < 0.001), with no significant difference in post-procedural gradient between groups (*p* = 0.257). Post-procedural LVEF did not change significantly from baseline within either group (TAx: 52.1 ± 7.3% to 49.7 ± 8.3%, *p* = 0.234; TF: 52.7 ± 11.9% to 51.0 ± 12.1%, *p* = 0.178), and post-procedural LVEF did not differ between groups (*p* = 0.059). ICU stay was shorter in the TAx group (median 2 [1–2] vs. 2 [1–3] days; *p* = 0.020). Hospital stay medians were identical between groups (5 [4–6] days for both; *p* = 0.005); the statistical significance of this comparison is attributable to outlier-driven mean prolongation in the TF group (mean: 5.0 ± 2.1 vs. 6.9 ± 5.8 days), including one patient with a 53-day admission. Pre- and post-procedural hemodynamic comparisons are illustrated in Figure 3.

### 3.3. Clinical Outcomes

Technical success was achieved in 94.0% of TAx patients and 96.0% of TF patients (OR 0.65 [95% CI 0.14–3.04]; *p* = 0.686). Device success rates were 94.0% and 99.0%, respectively (*p* = 0.108). Conversion to open surgery occurred in 2 TAx patients and 2 TF patients (OR 2.04, 95% CI 0.28–14.94; *p* = 0.601). Procedural myocardial infarction occurred in 0 TAx and 4 TF patients (OR 0.21, 95% CI 0.01–4.02; *p* = 0.302); odds ratios for these zero-event cells were estimated using the Haldane–Anscombe correction as detailed in Table 3. The VARC-3 early safety event composite rate was 16.0% in the TAx group versus 15.0% in the TF group, with no statistically significant difference between groups (OR 1.08, 95% CI 0.42–2.75; *p* = 1.000).

**Figure 4:**
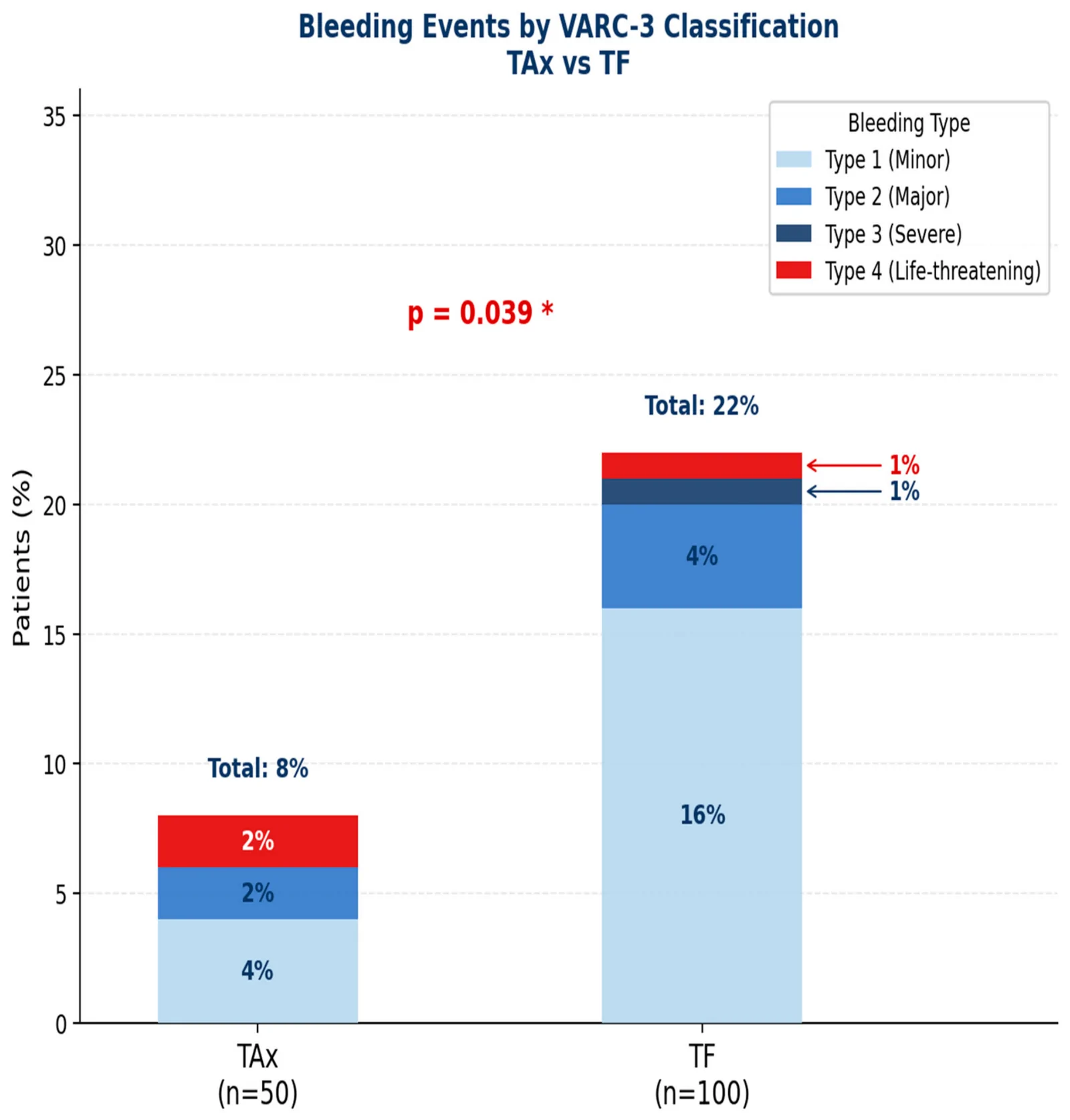
Stacked bar chart of bleeding events classified according to VARC-3 criteria in the TAx (n = 50) and TF (n = 100) groups. * Total any-bleeding rates were 8.0% in the TAx group and 22.0% in the TF group (*p* = 0.039). The difference was predominantly driven by Type 1 (minor) access-site bleeding events (TAx 4.0% vs. TF 16.0%). VARC-3, Valve Academic Research Consortium-3.

**Figure 5:**
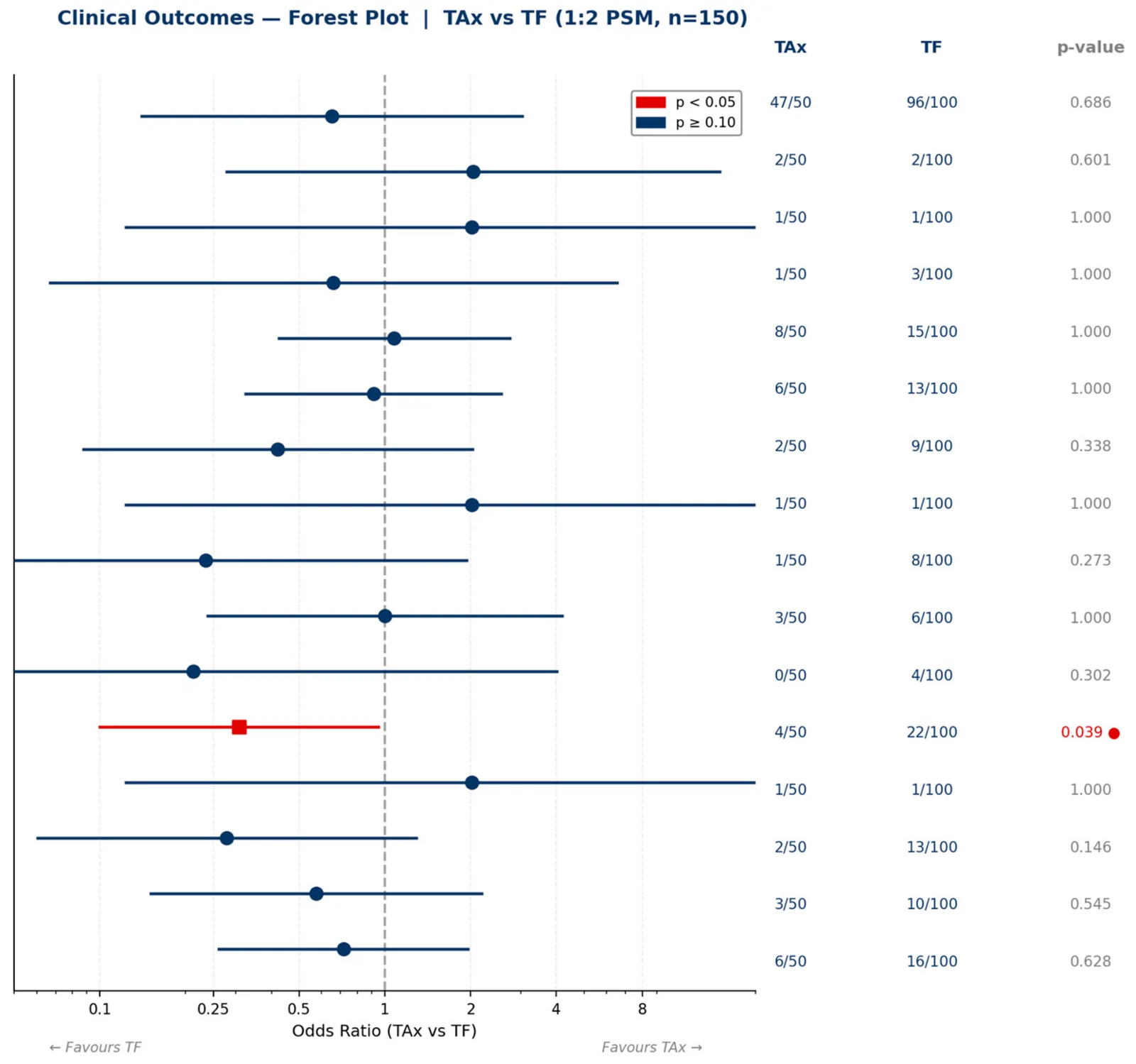
Forest plot of clinical outcomes comparing TAx versus TF access in the propensity score-matched cohort (n = 150). Odds ratios (squares) with 95% confidence intervals on a logarithmic scale are shown. Red filled squares indicate statistically significant differences (*p* < 0.05); orange indicates trend-level differences (*p* < 0.10); blue indicates non-significant results. The dashed vertical line denotes OR = 1.0. Patient event counts (TAx/TF) are displayed in the right-hand columns. VARC-3, Valve Academic Research Consortium-3; AR, aortic regurgitation; AKI, acute kidney injury; PPM, permanent pacemaker implantation.

The VARC-3 ESE composite occurred in 8 TAx patients (16.0%) and 15 TF patients (15.0%) (OR 1.08, 95% CI 0.42–2.75; *p* = 1.000). No statistically significant difference was detected; however, this does not establish equivalence given the small event counts and wide confidence intervals. Any bleeding (VARC-3) was significantly lower in the TAx group (4/50 [8.0%] vs. 22/100 [22.0%]; OR 0.31, 95% CI 0.10–0.95; *p* = 0.039). This result was confirmed by conditional logistic regression accounting for the matched 1:2 structure (OR 0.32, 95% CI 0.10–0.97; *p* = 0.043). The difference was predominantly driven by Type 1 (minor) access-site bleeding (TAx 4.0% vs. TF 16.0%), while Type 2–4 (clinically significant) bleeding rates were 4.0% vs. 6.0%, respectively (Figure 4). Given the borderline upper confidence interval (0.95 approaching the null) and the absence of multiplicity adjustment across multiple secondary comparisons, this result should be considered exploratory and hypothesis-generating. Thirty-day all-cause mortality, which encompasses all in-hospital deaths, was 6.0% vs. 10.0% (OR 0.57 [0.15–2.19]; *p* = 0.545), with no significant difference detected. Clinical outcomes are presented in Table 3 and Figure 5.

Rates of new permanent pacemaker implantation (12.0% vs. 13.0%; *p* = 1.000), moderate-to-severe paravalvular aortic regurgitation (4.0% vs. 9.0%; *p* = 0.338), stroke (2.0% vs. 1.0%; *p* = 1.000), neurological dysfunction without CNS injury (2.0% vs. 8.0%; *p* = 0.273), acute kidney injury > Stage 2 (6.0% vs. 6.0%; *p* = 1.000), and vascular reintervention (2.0% vs. 1.0%; *p* = 1.000) were comparable between groups. Procedure-related rehospitalization occurred in 4.0% of TAx patients and 13.0% of TF patients, a difference that did not reach statistical significance (*p* = 0.146).

### 3.4. Mortality and Survival

Intraprocedural mortality occurred in 1 TAx patient (2.0%) and 3 TF patients (3.0%), with no statistically significant difference between groups (*p* = 1.000). Thirty-day all-cause mortality, which encompasses all in-hospital deaths in this cohort, was 6.0% in the TAx group and 10.0% in the TF group (OR 0.57, 95% CI 0.15–2.19; *p* = 0.545). One-year all-cause mortality was 12.0% and 16.0%, respectively (OR 0.72, 95% CI 0.26–1.96; *p* = 0.628). While mortality rates were numerically lower in the TAx group at all time points, none of these differences reached statistical significance.

**Figure 6:**
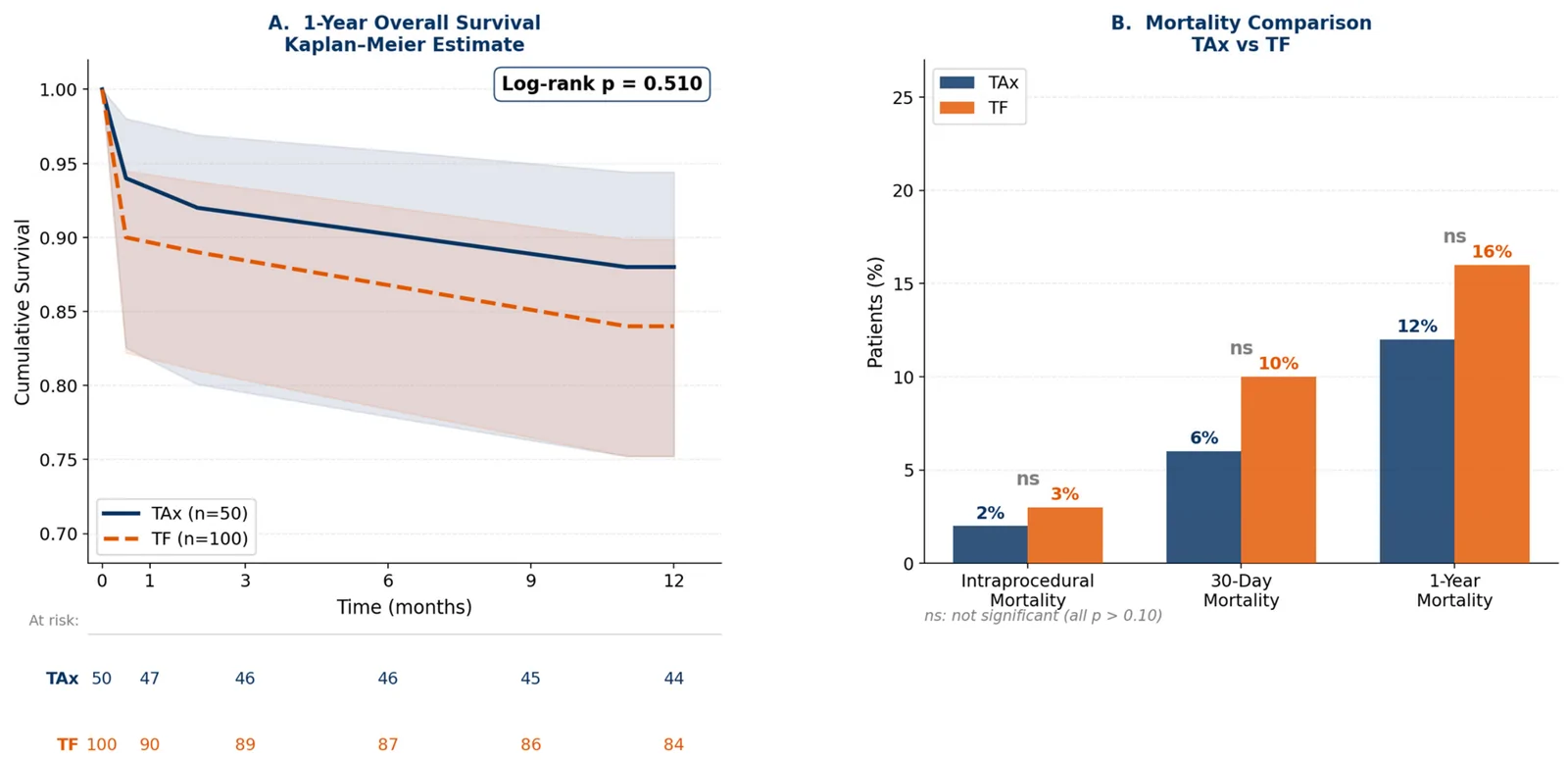
Panel (**A**): Kaplan–Meier curves for 1-year overall survival in the TAx (dark blue solid line) and TF (orange dashed line) groups. Shaded areas represent 95% confidence intervals. Numbers at risk are shown below the time axis. Log-rank *p* = 0.505. Panel (**B**): Bar chart comparing intraprocedural, 30-day, and 1-year all-cause mortality rates between the TAx and TF groups. ns, not significant. TAx, transaxillary access; TF, transfemoral access.

Kaplan–Meier estimated 1-year survival was 88.0% in the TAx group versus 84.0% in the TF group. The survival curves did not differ significantly between groups (log-rank *p* = 0.510). Mortality outcomes by time point are further compared in Figure 6.

## 4. Discussion

This propensity score-matched analysis, applying VARC-3 endpoint definitions throughout, yields three principal findings. First, TAx-TAVI achieves comparable technical success, device success, and VARC-3 ESE rates to TF-TAVI in a well-balanced matched cohort. Second, any bleeding (VARC-3) was significantly lower in the TAx group (OR 0.31, 95% CI 0.10–0.95; *p* = 0.039). Third, mortality, stroke, pacemaker implantation, renal outcomes, and hemodynamic efficacy were statistically equivalent between groups. These findings are broadly consistent with the existing comparative literature on axillary TAVI and extend current evidence to a contemporary VARC-3-based cohort with de novo ESE computation [7,8,15,16].

The significantly lower bleeding rate in the TAx group has a clear mechanistic rationale and direct clinical importance. Transfemoral access in patients with peripheral artery disease—the predominant indication for axillary access selection in our cohort (74% of TAx patients)—entails percutaneous puncture of calcified, tortuous femoral vessels at elevated risk for hematoma, dissection, and pseudoaneurysm [17,18]. We have previously characterized these vascular risk determinants in the transfemoral TAVI setting [6]. Notably, the TAx group utilized significantly larger sheath sizes than the TF group (15.9 ± 1.8 F vs. 14.7 ± 1.0 F; *p* < 0.001), yet achieved substantially lower bleeding rates. This apparent paradox underscores a fundamental principle: access-site bleeding in TAVI is determined predominantly by vascular anatomy and hemostatic control, rather than by sheath caliber alone. The axillary artery is characteristically spared from severe atherosclerosis and provides a surgically controlled environment through direct vessel exposure and purse-string hemostasis. Beyond the avoidance of heavily calcified iliofemoral segments, the anatomical positioning of the axillary artery offers a unique structural advantage: the first rib lies directly posterior to the proximal axillary segment, providing a rigid backstop that facilitates effective hemostasis through vessel loop control and suture compression, thereby minimizing the risk of occult hematomas—a catastrophic complication frequently encountered during complex transfemoral access management. We hypothesize that the purse-string suture technique contributed to reliable closure immediately after sheath removal, eliminating the uncertainty associated with percutaneous vascular closure devices in calcified femoral anatomy. That anatomically higher-risk TAx patients with larger sheath sizes nonetheless achieved a 14-percentage-point lower bleeding rate supports the hypothesis that controlled surgical axillary exposure mitigates hemorrhagic risk [6].

Our finding aligns with the meta-analysis by Al-Balah et al. (n = 2938 patients, nine studies), which found significantly lower major vascular complication rates with subclavian/axillary versus transfemoral access (OR 0.55, 95% CI 0.32–0.94) [7]. The propensity-matched Clermont-Ferrand comparison (n = 442, self-expanding valves) reported similar 30-day mortality between axillary and TF groups. Our results using predominantly balloon-expandable valves—supported by our institutional experience with the Meril [9] and Myval [10] systems via axillary access—suggest that the bleeding advantage of axillary access applies regardless of valve type, extending prior observations beyond the self-expanding platform. A recent single-center Turkish registry similarly confirmed the feasibility and safety of axillary access TAVI, supporting the generalizability of these findings to Turkish high-volume centers with established alternative access programs [19].

The comparable VARC-3 ESE rates (16.0% vs. 15.0%; *p* = 1.000) despite significantly lower any-bleeding in the TAx group reflect an important structural feature of the VARC-3 composite: Type 1 (minor) bleeding—the predominant driver of the bleeding difference (TAx 4.0% vs. TF 16.0%)—does not enter the ESE composite, which requires bleeding ≥ Type 2. This architectural nuance highlights the clinical value of reporting individual VARC-3 bleeding components alongside composite endpoints, as minor bleeding is independently associated with transfusion requirements, prolonged hospitalization, and patient discomfort even when excluded from composite safety measures.

The higher rate of balloon-expandable valve use in the TAx group (64.0% vs. 39.0%; *p* = 0.005) reflects our institutional preference and accumulated experience with balloon-expandable platforms via axillary access [9,10]. While historical axillary TAVI series predominantly utilized self-expanding platforms due to delivery system flexibility, our cohort demonstrates high-volume, safe utilization of balloon-expandable systems without compromised outcomes. The shorter stent frame design and precise, predictable radial expansion of modern balloon-expandable valves are hypothesized to confer potential mechanical advantages when navigating the acute angulation from the axillary trajectory into the aortic annulus: the millimetric ‘press-and-inflate’ deployment sequence may minimize the risk of deep valve implantation and eliminates the axial tension and kinking risk inherent to self-expanding nitinol frames advancing through the subclavian curvature. Furthermore, excellent coaxial alignment achieved with balloon-expandable systems in the axillary approach may explain the equivalent permanent pacemaker implantation rates observed between groups (12.0% vs. 13.0%; *p* = 1.000). Conduction disturbances are typically driven by excessive contact of the valve frame with the membranous septum due to deep implantation or loss of coaxiality; the millimetric control of balloon-expandable deployment mitigates these risks even in the angulated axillary geometry. Comparable post-procedural hemodynamics (mean gradient 10.3 ± 2.0 vs. 10.4 ± 4.3 mmHg; *p* = 0.257) confirm equivalent valve function independent of access route [20].

A specific clinical concern in the axillary approach deserves attention given that 32.0% of our matched cohort carried a history of prior sternotomy. In patients with a patent left internal mammary artery (LIMA) graft, sheath placement through the left axillary artery poses a theoretical risk of intraprocedural graft hypoperfusion or LIMA-steal syndrome secondary to sheath-induced flow reduction at the subclavian ostium. In our institutional protocol, a patent LIMA was not regarded as an absolute contraindication to axillary access provided that the vessel diameter exceeded 7 mm and the artery was free of significant atherosclerotic disease. MDCT-based measurement of the distance from the LIMA ostium to the planned puncture site guided access planning in all such patients. The absence of periprocedural myocardial infarction in the TAx group (0.0% vs. 2.0%; *p* = 0.553) confirms that this selective approach—combining anatomical MDCT criteria with short-profile delivery systems—safely mitigates the risk of graft-territory ischemia.

The lower use of local anesthesia in the TAx group (78.0% vs. 95.0%; *p* = 0.003) reflects the learning curve of our axillary TAVI program over the 2016–2024 study period. Early procedures were performed under general anesthesia; local anesthesia with conscious sedation was progressively adopted as operator confidence and Heart Team protocols matured. This institutional learning curve—spanning surgical cutdown standardization, purse-string suture mastery, and valve deployment optimization via the axillary trajectory—reflects a broader principle: axillary TAVI should not be viewed merely as a rescue strategy for failed transfemoral access, but rather as a proactive, co-primary alternative in experienced centers with high-volume surgical backup and structured multidisciplinary expertise. Transfemoral TAVI, by contrast, was performed under local anesthesia throughout the study period.

The systematic preference for the left axillary artery in our program is anatomically intentional. The left subclavian–axillary trajectory provides a more direct, coaxial, and less acutely angled route into the ascending aorta and aortic annulus compared to the right-sided approach, which requires navigating the brachiocephalic trunk and introduces a more perpendicular angulation that increases the risk of valve malalignment and large-bore catheter kinking. Furthermore, right-sided access necessitates crossing the brachiocephalic bifurcation, carrying a theoretically higher risk of carotid atheroembolism during large-bore instrumentation. For these anatomical and safety reasons, left axillary access represents the preferred default in experienced centers.

Mortality outcomes are consistent with contemporary registry data for comparable TAVI populations [3,4,21,22]. Importantly, despite the TAx group carrying a fundamentally higher baseline surgical risk profile—as evidenced by a mean STS score more than double that of the TF cohort (8.8 ± 3.2% vs. 3.8 ± 2.4%; *p* < 0.001)—long-term survival and early safety composite rates remained clinically identical. This STS score differential reflects the inherently high-risk nature of the TAx population: beyond the 74% prevalence of peripheral artery disease (which independently elevates STS-predicted risk through its association with multivessel atherosclerosis, renal insufficiency, and reduced functional reserve), the TAx cohort also carried higher rates of prior sternotomy (32%), coronary artery disease, and complex comorbidity patterns that cumulatively drive STS score elevation. Thirty-day mortality was 6.0% (TAx) versus 10.0% (TF) (*p* = 0.545), and 1-year Kaplan–Meier survival was 88.0% versus 84.0% (log-rank *p* = 0.510). This pivotal observation suggests that the axillary approach may act as a clinical risk-equalizer, effectively neutralizing the expected excess mortality driven by severe peripheral vasculopathy and comorbidity burden in patients disqualified from the transfemoral route. The substantially higher STS scores in the TAx group were deliberately excluded from the PSM model given their mechanistic link to access indication; the absence of a statistically significant mortality difference despite this risk differential may partly reflect insufficient statistical power (Type II error), and larger multicenter studies are needed to determine whether the observed numerical survival advantage in the TAx group represents a true clinical signal.

Neurovascular safety represents a further benchmark for alternative access selection. Navigating large-bore delivery systems through the subclavian artery raises theoretical concerns for atheroembolism into the left vertebral artery, potentially causing posterior circulation stroke. Our institutional approach specifically targets the distal subclavian–proximal axillary segment for vascular access, a site anatomically distal to the origin of the vertebral artery from the subclavian trunk. This critical detail—confirmed on MDCT prior to each procedure—means that sheath placement and subsequent valve delivery system manipulation occur entirely distal to the vertebral ostium, effectively eliminating the mechanism for vertebral artery embolization. Our data corroborate this safety rationale: stroke occurred in only 1 TAx patient (2.0%) versus 1 TF patient (1.0%), with no statistically significant difference (*p* = 1.000), demonstrating a neurologically safe profile for the distal axillary access strategy.

Regarding hospital and intensive care stay, the two groups had identical median values (hospital stay: 5 [4–6] days for both; ICU stay: 2 days for both), confirming no clinically meaningful difference in typical recovery duration. The significant *p*-values (*p* = 0.005 and *p* = 0.020 respectively) are driven by outlier-prolonged admissions in the TF group. A clinical explanation for the TAx group’s recovery efficiency lies in the surgical nature of the axillary approach: unlike transfemoral TAVI, which requires prolonged absolute supine bed rest to prevent femoral access-site disruption, pseudoaneurysm formation, or hematoma expansion, the purse-string sutured axillary wound supports early mobilization. Patients are able to achieve upright posture and begin ambulation within hours of the procedure, reducing immobilization-related risks including micro-atelectasis, hypostatic pneumonia, venous thromboembolism, and functional deconditioning. This early ambulation advantage is hypothesized to contribute to the shorter ICU monitoring requirements observed in the TAx group, though this mechanism was not directly tested in the current study.

### Limitations

This study has important limitations. First, the retrospective single-center design introduces potential selection, temporal, and ascertainment biases. Most critically, 2098 of 2389 screened patients (87.7%) were excluded: approximately 1650 due to insufficient follow-up duration (<12 months), and approximately 448 due to incomplete baseline covariate data precluding propensity score estimation. This high exclusion rate constitutes a substantial source of potential complete-case bias. A systematic comparison of included versus excluded patients was not feasible, as excluded patients lacked the complete covariate profiles required for such analysis; whether the included population systematically differed from the excluded population in unmeasured prognostic characteristics cannot be determined. The TAx group carried a substantially higher burden of peripheral artery disease (74%) and STS score (8.8 ± 3.2% vs. 3.8 ± 2.4%), and while PSM balanced all nine matched covariates, STS score was deliberately excluded from the matching model owing to its mechanistic link to access indication. Residual confounding from this risk differential and from unmeasured variables (CT-derived vascular anatomy, frailty, operator experience) cannot be excluded.

Second, the relatively modest sample size, while sufficient for primary endpoint analysis, limits statistical power for rarer secondary outcomes. Confidence intervals around odds ratios for endpoints such as stroke, valve embolization, and vascular reintervention are wide, and the absence of statistically significant differences for these outcomes should not be interpreted as equivalence.

Third, outcome analyses were primarily conducted using Fisher’s exact test and the Mann–Whitney U test, which do not fully account for the matched 1:2 structure. Conditional logistic regression was performed as a pre-specified sensitivity analysis and confirmed consistency of the primary bleeding finding (OR 0.32, 95% CI 0.10–0.97; *p* = 0.043). Fourth, VARC-3 endpoint definitions were retrospectively applied across the full 2016–2024 study period; independent adjudication by a clinical events committee was not performed. The ESE composite was computed de novo from individual component data to minimize retrospective misclassification. Fifth, the 9-year study period encompasses substantial evolution in valve platforms, delivery systems, and clinical practice. A formal era-stratified sensitivity analysis was not feasible given the small TAx sample (n = 50); temporal confounding therefore cannot be excluded.

Fourth, EuroSCORE I and II data were unavailable for TF patients in our institutional database, precluding their inclusion in the propensity score model. Although STS score was available and used in descriptive analysis, the absence of European risk stratification data limits risk-adjusted comparisons across groups.

Fifth, survival analysis was based on monthly mortality timing data rather than exact event dates, which may introduce minor imprecision in Kaplan–Meier estimates. However, given the 12-month follow-up horizon and the availability of timing data for all decedents, the impact on curve morphology and log-rank test results is expected to be minimal.

Finally, the observational nature of the study and the indication-based access route selection preclude causal inference. The propensity-matched design reduces—but does not eliminate—confounding, and the present findings should be interpreted as hypothesis-generating observational evidence. Randomized controlled trial data comparing axillary and transfemoral TAVI access remain the methodological gold standard but are logistically challenging given the indication-driven nature of alternative access selection in clinical practice.

## 5. Conclusions

In this propensity score-matched, VARC-3-based single-center analysis, transaxillary access for TAVI demonstrated comparable technical success, VARC-3 early safety event rates, post-procedural hemodynamic efficacy, and short- and long-term mortality outcomes to the transfemoral approach. Any bleeding (VARC-3) was significantly lower in the TAx group ((OR 0.31 [0.10–0.95]; *p* = 0.039), confirmed by conditional logistic regression (OR 0.32 [0.10–0.97]; *p* = 0.043). This result was predominantly driven by Type 1 minor access-site bleeding and should be considered an exploratory, hypothesis-generating finding given the borderline confidence interval, small sample size, and absence of multiplicity correction. These findings support surgically controlled transaxillary access as a feasible and safe alternative in carefully selected patients with unfavorable iliofemoral anatomy at experienced centers with established alternative access programs. Prospective multicenter registries with standardized VARC-3 endpoint reporting and formal matched-analysis methodology are needed to confirm these observations.

## Figures and Tables

**Table 1:** Baseline characteristics of the propensity score-matched cohort.

Variable	TAx (n = 50)	TF (n = 100)	*p*-Value
Demographics			
Age, years	79.0 ± 5.8	79.1 ± 5.4	0.865
Male sex, n (%)	27 (54.0)	56 (56.0)	0.863
BMI, kg/m^2^	28.4 ± 4.8	28.7 ± 5.1	0.714
Comorbidities			
Hypertension, n (%)	39 (78.0)	79 (79.0)	1.000
Diabetes mellitus, n (%)	17 (34.0)	37 (37.0)	0.865
Coronary artery disease, n (%)	31 (62.0)	65 (65.0)	0.849
Previous myocardial infarction, n (%)	10 (20.0)	17 (17.0)	0.820
Previous PCI, n (%)	16 (32.0)	26 (26.0)	0.529
Previous CABG, n (%)	6 (12.0)	12 (12.0)	1.000
Prior sternotomy, n (%)	16 (32.0)	32 (32.0)	1.000
Permanent pacemaker, n (%)	5 (10.0)	12 (12.0)	0.779
Previous stroke, n (%)	3 (6.0)	5 (5.0)	1.000
Peripheral artery disease, n (%)	37 (74.0)	24 (24.0)	<0.001
COPD, n (%)	14 (28.0)	26 (26.0)	0.847
Chronic kidney disease, n (%)	7 (14.0)	15 (15.0)	1.000
Porcelain aorta, n (%)	5 (10.0)	3 (3.0)	0.118
Malignancy, n (%)	6 (12.0)	11 (11.0)	1.000
Risk Score			
STS Score, %	8.8 ± 3.2	3.8 ± 2.4	<0.001
Echocardiography and Anatomy			
LVEF, %	52.1 ± 9.2	52.7 ± 10.1	0.654
Aortic valve area, cm^2^	0.6 ± 0.2	0.7 ± 0.2	0.004
Mean aortic gradient, mmHg	46.2 ± 14.3	48.5 ± 15.1	0.282
Access vessel diameter, mm	6.8 ± 1.1	8.1 ± 1.4	<0.001

BMI, body mass index; CABG, coronary artery bypass grafting; COPD, chronic obstructive pulmonary disease; CKD, chronic kidney disease; LVEF, left ventricular ejection fraction; PCI, percutaneous coronary intervention; TAx, transaxillary access; STS, Society of Thoracic Surgeons; TF, transfemoral access. Values are mean ± SD or n (%).

**Table 2:** Procedural characteristics and post-procedural outcomes.

Variable	TAx (n = 50)	TF (n = 100)	*p*-Value
Procedural Details			
Balloon-expandable valve, n (%)	32 (64.0)	39 (39.0)	0.005
Local anesthesia, n (%)	39 (78.0)	95 (95.0)	0.003
Sheath size, F	15.9 ± 1.8	14.7 ± 1.0	<0.001
Post-Procedural Hemodynamics			
Post-proc. LVEF, %	49.7 ± 8.3	51.0 ± 12.1	0.059 †
Post-proc. mean aortic gradient, mmHg	10.3 ± 2.0	10.4 ± 4.3	0.257
Hospital Course			
ICU stay, days, median [IQR]	2 [1–2]	2 [1–3]	0.020 ‡
Hospital stay, days, median [IQR]	5 [4–6]	5 [4–6]	0.005 ‡

F, French; ICU, intensive care unit; IQR, interquartile range; LVEF, left ventricular ejection fraction; TAx, transaxillary access; TF, transfemoral access. Values are mean ± SD or median [IQR]. † Post-procedural LVEF: within-group change non-significant (TAx *p* = 0.234; TF *p* = 0.178); *p*-value shown is between-group post-procedural comparison. ‡ Medians identical between groups; statistically significant *p*-values reflect outlier-driven prolongation in the TF group (mean: 5.0 ± 2.1 vs. 6.9 ± 5.8 days for hospital stay) rather than a meaningful difference in typical recovery duration.

**Table 3:** Clinical outcomes in the propensity score-matched cohort.

Outcome	TAx (n = 50)	TF (n = 100)	OR (95% CI)	*p*-Value
Procedural Outcomes				
Technical success, n (%)	47 (94.0)	96 (96.0)	0.65 [0.14–3.04]	0.686
Device success, n (%)	47 (94.0)	99 (99.0)	0.16 [0.01–1.35]	0.108
Conversion to open surgery, n (%) §	2 (4.0)	2 (2.0)	2.04 [0.28–14.94]	0.601
Valve embolization, n (%)	1 (2.0)	1 (1.0)	2.01 [0.12–33.5]	1.000
Intraprocedural mortality, n (%)	1 (2.0)	3 (3.0)	0.66 [0.07–6.52]	1.000
30-Day Outcomes (VARC-3)				
Early safety event (VARC-3) *, n (%)	8 (16.0)	15 (15.0)	1.08 [0.42–2.75]	1.000
New permanent pacemaker, n (%)	6 (12.0)	13 (13.0)	0.91 [0.32–2.57]	1.000
Mod-severe paravalvular AR, n (%)	2 (4.0)	9 (9.0)	0.42 [0.09–2.02]	0.338
Stroke, n (%)	1 (2.0)	1 (1.0)	2.01 [0.12–33.5]	1.000
Neurological dysfunction (no CNS injury), n (%)	1 (2.0)	8 (8.0)	0.24 [0.03–1.96]	0.273
AKI > Stage 2 (KDIGO), n (%)	3 (6.0)	6 (6.0)	1.00 [0.24–4.22]	1.000
Procedural MI, n (%) §	0 (0.0)	4 (4.0)	0.21 [0.01–4.02]	0.302
Any bleeding (VARC-3) †, n (%)	4 (8.0)	22 (22.0)	0.31 [0.10–0.95]	0.039
CLR sensitivity analysis			0.32 [0.10–0.97]	0.043
Vascular reintervention, n (%)	1 (2.0)	1 (1.0)	2.01 [0.12–33.5]	1.000
Rehospitalization (procedure-related), n (%)	2 (4.0)	13 (13.0)	0.28 [0.06–1.30]	0.146
30-Day mortality (incl. in-hospital), n (%)	3 (6.0)	10 (10.0)	0.57 [0.15–2.19]	0.545
Bleeding—VARC-3 Classification				
Type 1 (minor): bruising, hematoma < 5 cm	2 (4.0)	16 (16.0)	—	—
Type 2 (major): hematoma ≥ 5 cm or transfusion < 2 units	1 (2.0)	4 (4.0)	—	—
Type 3 (severe): Hgb drop ≥ 3 g/dL or transfusion ≥ 2 units	0 (0.0)	1 (1.0)	—	—
Type 4 (life-threatening): fatal or critical organ	1 (2.0)	1 (1.0)	—	—
Vascular Complications—VARC-3				
Minor, n (%)	1 (2.0)	0 (0.0)	—	—
Major, n (%)	1 (2.0)	1 (1.0)	—	—
Long-Term Outcomes				
1-Year mortality, n (%)	6 (12.0)	16 (16.0)	0.72 [0.26–1.96]	0.628
1-Year survival (Kaplan–Meier)	88.0%	84.0%	—	0.510 ‖

AKI, acute kidney injury (KDIGO criteria per VARC-3); AR, aortic regurgitation; CI, confidence interval; CLR, conditional logistic regression (cluster = Match ID); CNS, central nervous system; KDIGO, Kidney Disease: Improving Global Outcomes; MI, myocardial infarction; OR, odds ratio; TAx, transaxillary access; TF, transfemoral access; VARC-3, Valve Academic Research Consortium-3. * ESE composite (VARC-3): 30-day mortality, stroke, bleeding ≥ Type 2, AKI >Stage 2, major vascular complication, major cardiac structural complication, valve embolization, vascular reintervention. † Primary outcome: Fisher’s exact *p* = 0.039; CLR sensitivity *p* = 0.043 (OR 0.32 [0.10–0.97]). Given the borderline CI and exploratory multiple comparisons, this result is hypothesis-generating. See Supplementary Table S1 for CLR results for all outcomes. § OR estimated using Haldane–Anscombe correction (zero events in one group) and corrected estimates are noted. VARC-3 Bleeding types: Type 1 (minor): superficial bruising or hematoma < 5 cm; Type 2 (major): hematoma ≥ 5 cm, or transfusion < 2 units, or requiring intervention; Type 3 (severe): Hgb drop ≥ 3 g/dL, or transfusion ≥ 2 units, or critical organ bleeding; Type 4 (life-threatening): fatal or requiring urgent intervention. ‖ Log-rank *p*-value.

## Data Availability

The raw data will be made available by the corresponding author on request.

## Supplementary Materials

The following supporting information can be downloaded at: https://www.mdpi.com/article/10.3390/diagnostics16172855/s1, Supplemantary Table S1: Comparison of Fisher’s Exact Test and Conditional Logistic Regression Results for All Binary Outcomes in the Propensity Score-Matched Cohort (n = 150; 50 TAx, 100 TF).

## Abbreviations

The following abbreviations are used in this manuscript:

TAx	Transaxillary access
TAVI	Transcatheter aortic valve implantation
TF	Transfemoral access
