# Supplementary material for: Transaxillary Versus Transfemoral Access for Transcatheter Aortic Valve Implantation: A Propensity Score-Matched Comparison of Clinical Outcomes Using VARC-3 Endpoints
Source: Diagnostics (Basel). 2026 Sep 5;16(17):2855. doi: 10.3390/diagnostics16172855 (PMC13564691; doi:10.3390/diagnostics16172855)
Supplement: Supplementary file 1 [file diagnostics-16-02855-s001.zip › diagnostics-4469212-supplementary.pdf]

Supplementary Table S1. Comparison of Fisher's Exact Test and Conditional Logistic Regression Results for All Binary Outcomes in the Propensity Score-Matched Cohort (n = 150; 50 TAx, 100 TF).

Outcome	TAx n/N (%)	TF n/N (%)	Fisher's Exact OR (95% CI)	Fisher's p-value	Conditional Logistic Regression OR (95% CI)	CLR p-value
Technical Success	47 (94%)	96 (96%)	0.65 [0.14–3.04]	0.686	0.67 [0.15–2.98]	0.596
Conversion to Open Surgery	2 (4%)	2 (2%)	2.04 [0.28–14.94]	0.601	2.04 [0.28–14.94]	0.488
Valve Embolization	1 (2%)	1 (1%)	2.02 [0.12–32.99]	1.000	2.00 [0.13–32.02]	0.623
Intraprocedural Mortality	1 (2%)	3 (3%)	0.66 [0.07–6.51]	1.000	0.67 [0.07–6.41]	0.726
Early Safety Event (VARC-3)	8 (16%)	15 (15%)	1.08 [0.42–2.75]	1.000	1.09 [0.41–2.88]	0.867
New Permanent Pacemaker	6 (12%)	13 (13%)	0.91 [0.32–2.56]	1.000	0.91 [0.32–2.60]	0.860
Mod-Severe Paravalvular AR	2 (4%)	9 (9%)	0.42 [0.09–2.03]	0.338	0.44 [0.10–2.06]	0.300
Stroke	1 (2%)	1 (1%)	2.02 [0.12–32.99]	1.000	2.00 [0.13–32.02]	0.623
Neurological Dysfunction	1 (2%)	8 (8%)	0.23 [0.03–1.93]	0.273	0.25 [0.03–2.00]	0.191
AKI > Stage 2 (KDIGO)	3 (6%)	6 (6%)	1.00 [0.24–4.18]	1.000	1.00 [0.23–4.35]	1.000
Procedural MI	0 (0%)	4 (4%)	0.21 [0.01–4.02]	0.302	NC	0.784
Any Bleeding (VARC-3)	4 (8%)	22 (22%)	0.31 [0.10–0.95]	0.039 *	0.32 [0.10–0.97]	0.043 *
Vascular Reintervention	1 (2%)	1 (1%)	2.02 [0.12–32.99]	1.000	2.00 [0.13–32.02]	0.623
Rehospitalization	2 (4%)	13 (13%)	0.28 [0.06–1.29]	0.146	0.29 [0.06–1.32]	0.108
30-Day Mortality (incl. in-hospital)	3 (6%)	10 (10%)	0.57 [0.15–2.19]	0.545	0.58 [0.15–2.19]	0.419
1-Year Mortality	6 (12%)	16 (16%)	0.72 [0.26–1.96]	0.628	0.69 [0.24–1.98]	0.495

*OR, odds ratio; CI, confidence interval; CLR, conditional logistic regression; TAx, transaxillary access; TF, transfemoral access; VARC-3, Valve Academic Research Consortium-3; AKI, acute kidney injury; PPM, permanent pacemaker; AR, aortic regurgitation; NC, not computable. Fisher's exact test uses unmatched four-fold-table OR and does not account for the matched 1:2 cluster structure. CLR uses Match ID as the cluster variable to account for the matched design. For cells with zero events, the Haldane–Anscombe correction (addition of 0.5 to all cells) was applied before OR computation. Highlighted rows (yellow): statistically significant result in either analysis. * p < 0.05.*
